# Dietary supplementation with a complex of cinnamaldehyde, carvacrol, and thymol negatively affects the intestinal function in LPS-challenged piglets

**DOI:** 10.3389/fvets.2023.1098579

**Published:** 2023-03-30

**Authors:** Yanyan Zhang, Qian Li, Zhongxing Wang, Yi Dong, Dan Yi, Tao Wu, Lei Wang, Di Zhao, Yongqing Hou

**Affiliations:** Engineering Research Center of Feed Protein Resources on Agricultural By-Products, Ministry of Education, Wuhan Polytechnic University, Wuhan, China

**Keywords:** piglet, a complex of cinnamaldehyde, carvacrol and thymol, lipopolysaccharide, intestinal function, immune stress response

## Abstract

**Background:**

The effects of cinnamaldehyde, carvacrol and thymol complex (CCT) on the growth performance and intestinal function of piglets challenged with lipopolysaccharide (LPS) were determined. Colistin sulphate (CS) was as a positive control.

**Method:**

Piglets (*n* = 24, 32 days of age) were allocated to four treatments: Control group (fed basal diet), LPS group (fed basal diet), CS+LPS group (fed basal diet + 50 mg/kg CS), and CCT+LPS group (fed basal diet + 50 mg/kg CCT).

**Results:**

Results showed that diarrhea rates of piglets were significantly reduced by CCT and CS supplementation respectively. Further research showed that CS supplementation tended to improve the intestinal absorption function in LPS-challenged piglets. Moreover, CS supplementation significantly reduced the contents of cortisol in blood and malondialdehyde in the duodenum and the activities of inducible nitric oxide synthase in the duodenum and ileum and total nitric oxide synthase in the ileum in LPS-challenged piglets. CS supplementation significantly increased the activities of sucrase in the ileum and myeloperoxidase in the jejunum in LPS-challenged piglets. CS supplementation significantly alleviated the reduced mRNA levels of immune-related genes (IL-4, IL-6, IL-8, IL-10) in mesenteric lymph nodes and jejunum and mucosal growth-related genes (IGF-1, mTOR, ALP) in LPS-challenged piglets. These results suggested that CS supplementation improved the intestinal function in LPS-challenged piglets by improving intestinal oxidative stress, immune stress, and absorption and repair function. However, although CCT supplementation improved oxidative stress by reducing (*p* < 0.05) the content of malondialdehyde and the activity of nitric oxide synthase in the duodenum, CCT supplementation tended to aggravate the intestinal absorption dysfunction in LPS-challenged piglets. Furthermore, compared with the control and LPS groups, CCT supplementation remarkably elevated the content of prostaglandin in plasma and the mRNA levels of pro-inflammatory factor IL-6 in mesenteric lymph nodes and jejunum, and reduced the activity of maltase in the ileum in LPS-challenged piglets. These results suggested that CCT supplementation had a negative effect on intestinal function by altering intestinal immune stress response and reducing disaccharidase activity in LPS-challenged piglets.

**Conclusions:**

Compared to CS, CCT supplementation exhibited a negative effect on intestinal function, suggesting whether CCT can be as an effective feed additive still needs further study.

## Introduction

Piglet feeding is one of the most critical parts of modern pig industry (1). However, piglets have a low immunity and an underdeveloped intestinal system, and piglets are prone to various stress responses (2). Moreover, the changes of dietary from easily digestible milk to less digestible solid feeds led to intestinal dysfunction in piglets and increased sensitivity of piglets to pathogenic microorganisms (3). Therefore, weaning leads to high morbidity and mortality in piglets. Although the use of antibiotics can significantly reduce the morbidity and mortality of piglets, a series of side effects such as drug resistance and drug residues also appear (4). The European Union and other countries banned the use of antibiotic growth-promoting agents in 2006 (5). Subsequently, China banned the use of antibiotic growth-promoting agents in 2020 (6). Faced with a series of health problems in piglet farming after the ban on antibiotics, it is crucial for swine industry to develop high-quality, safe and efficient additives that can replace antibiotics.

In recent years, natural plant extracts have been widely studied as potential feed additives (7). Among them, plant essential oils were the most widely studied and used (8). Previous studies have shown that plant essential oils supplemented in diet can enhance the growth performance of piglets by increasing feed intake, promoting digestion, exerting antibacterial, anti-inflammatory and antioxidant effects, and enhancing immune functions (9, 10).

Oleum cinnamomi, oregano and thyme oil were widely studied in feed production. Oleum cinnamomi has been reported to exhibit potential antibacterial activity and metabolic-modulating effects (11, 12) And Oleum cinnamomi can protect the intestine from damage under conditions of oxidative stress, inflammation, and infections (13). Cinnamaldehyde as a potent inhibitor is the predominant bioactive compound in oleum cinnamomi, which can inhibit filamentous molds, bacteria, and yeast (14). Cinnamaldehyde has been regarded as a potent antibiotic alternative in livestock production. Previous studies showed that cinnamaldehyde increased the antioxidant activity of rat kidneys by increasing the activity of the antioxidant enzyme (15). Cinnamaldehyde added to the diet may reduce the effects of stress and promote intake in feedlot cattle particularly early in the feeding period (16). Cinnamaldehyde also plays an anti-inflammatory role in *Helicobacter pylori*-induced gastric inflammation (17). Oregano has also been reported to exhibit potential antibacterial activity and antioxidant properties (18). Carvacrol, as the predominant bioactive in oregano, has antimicrobial, antioxidant, anti-inflammatory, anti-osteoclastic, and anti-diabetic properties combined with magnolol (19). Thyme oil has also been reported to exhibit potential antibacterial activity (18). Carvacrol and thymol, as the predominant bioactive in thyme oil, can improve absorption capacity by enhancing the digestive enzyme activity. And carvacrol and thymol also contribute to the relief from pathogen pressure by stimulating intestinal mucus production (20). Furthermore, previous studies have been reported that carvacrol and thymol supplementation promoted growth and improved meat and egg quality through metabolic-modulating, anti-oxidative, anti-inflammatory, and antimicrobial effects in poultry production (21). These 3 plant extracts have been reported to alter membrane permeability to hydrogen ions and reduce the number of pathogens in the intestinal tract (22).

In view of the good effect of cinnamaldehyde, carvacrol and thymol in metabolic-modulating, anti-oxidative, anti-inflammatory, and antimicrobial effects, whether the combination of cinnamaldehyde, carvacrol and thymol can better promote animal growth has not been studied yet. To evaluate the effects of dietary supplementation with cinnamaldehyde, carvacrol and thymol complex (CCT) on growth performance and intestinal function in weaned piglets, a model of piglet intestinal injury induced by lipopolysaccharide (LPS) stimulation was used in this study. LPS, as an immune stressor, can disrupt bowel function by affecting the expression of genes related to intestinal immune responses, growth, absorption, mucosal energy metabolism, and mucosal barrier function (23). The model of intestinal injury induced by LPS stimulation has been widely used to screen antibiotic substitutes (24–27). To further evaluate whether CCT can be used as a potential antibiotic substitute in feed, colistin sulfate (CS), as an antibiotic growth promoter which has been extensively used as a feed additive to reduce pathogen infections and improve growth performance after weaning (28, 29), was served as a positive control in this study.

Therefore, this study investigated the effects of dietary supplementation with CCT on the growth performance and intestinal function in weaned piglets challenged by LPS.

## Materials and methods

### Animals experiment design

In the present study, animal experiment was approved by the Institutional Animal Care and Use Committee at Wuhan Polytechnic University (2014-0514, May 10, 2014). All animal experiments were performed following the guidelines of the Research Ethics Committee of the College of Animal Science and Nutritional Engineering, Wuhan Polytechnic University. The animal experiments were also conducted in accordance with relevant guidelines and regulations. Twenty-four crossbred healthy female piglets (Duroc × Landrace × Yorkshire) were weaned after 21 days after birth. After 11 days of environmental adaptation, piglets (32 days old, 7.29 ± 0.77 kg) were housed individually in stainless steel metabolic cages (1.20 × 1.10m^2^), and environmental temperature was maintained at 22–25°C (30). Food and water can be freely obtained by piglets. The corn and soybean meal-based diet was formulated to meet National Research Council's (31) recommended requirements for all nutrients [National Research Council. Nutrient Requirements of pigs. National Academic Press: Washington, DC (2012)]. During the 11-day adaptation period, all piglets were fed the basal diet.

At 32 days old (set as the day 0 of the experiment), all piglets were divided freely into one of the four groups: (1) Control group (fed the basal diet and sterile saline was injected intraperitoneally); (2) LPS group (fed the basal diet and LPS was injected intraperitoneally); (3) Colistin sulfate (CS)+LPS group (fed the basal diet supplemented with 50 mg/kg CS and LPS was injected intraperitoneally); (4) cinnamaldehyde, carvacrol and thymol complex (CCT)+LPS group (fed basal diet supplemented with 50 mg/kg CCT complex and LPS was injected intraperitoneally).

The trial period was 21 days (day 0 to 20 days). The growth performance of piglets was statistically analyzed by recording the body weight (BW), feed intake and diarrhea rates (DR). The blood samples were gathered from the anterior vena cava of 12-h-fasted pigs into heparinized vacuum tubes (Becton Dickinson Vacutainer System) on day 21 of the trial, the collected blood samples were used for hematological evaluation and separating plasma by centrifuging (3,500 × g for 10 min at 4°C) (13). Subsequently, overnight fasted piglets in the LPS, CS+LPS and CCT+LPS groups were intraperitoneally injected with LPS (100 μg/kg BW, *Escherichia coli* serotype 055: B5; Sigma Chemical Inc., St. Louis, MO, USA), whereas those in the control group were intraperitoneally injected with the same volume of sterile saline (30). To determine intestinal absorptive function, all piglets was orally administrated D-xylose with the dose of 0.1 g/kg BW at 2 h post LPS challenge (32). At 3h after LPS challenge, blood samples were gathered and stored at −80°C until analysis. At 6h after LPS challenge, all piglets were killed under anesthesia with an intravenous injection of pentobarbital sodium (50 mg/kg BW), and then intestinal samples were collected.

In addition, cinnamaldehyde (St. Louis, MO, USA; Cat. W228613; CAS.8007-80-5), carvacrol (CAS.499-75-2) and thymol (CAS.89-83-8) were purchased from Sigma-Aldrich Chemicals. The dosage of 50 mg/kg CCT was chosen according to our previous study by Wang et al. (13). The proportion of the complex is determined according to the content of the main effective substances in Oleum cinnamomi, oregano oil and thyme oil (1:1:1).

### Collection of intestinal samples

The whole gastrointestinal tracts of piglets were immediately exposed by quickly opening abdomen from the sternum to the pubis (32). The small intestine dissected free of the mesentery was placed on a stainless steel tray (< 0°C). The different intestinal segments (10 cm) were collected from the distal duodenum, mid-jejunum and mid-ileum. And then the collected segments were opened longitudinally with scissors, and the exposed intestinal cavity surface was flushed with ice-cold PBS (30). A sterile glass microscope slide was used to scrape mucosal samples at 4°C, and then the scraped mucosal was rapidly frozen in liquid nitrogen and stored at −80°C until analysis. After piglets were killed, all samples were collected within 15 min.

### Hematology measurement

The whole blood samples were analyzed by the ADVIA 2120i Hematology System (Siemens Healthcare Diagnostics, Deerfield, Illinois, USA) to determine hematology including the white blood cell (WBC), red blood cell (RBC), platelet (PLT), plateletcrit (PCT), hemoglobin (HGB), hematocrit (HCT), red blood cell distribution width (RDW), mean corpuscular volume (MCV), mean corpuscular hemoglobin (MCH), mean corpuscular hemoglobin concentration (MCHC), and mean platelet volume (MPV).

### Plasma biochemistry

Plasma biochemical parameters, such as aspartate transaminase (AST), total bilirubin (TBIL), alanine transaminase (ALT), triacylglycerol (TG), blood urea nitrogen (BUN), and alkaline phosphatase (ALP), were determined by an automatic biochemical analyzer (HITACHI 7020, Japan).

### D-xylose in plasma

The plasma D-xylose was measured as described by Yi et al. (33). Fifty microliters of plasma was added to 5 mL of the phloroglucinol color reagent solution (Sigma Chemical Inc., St. Louis, MO, USA) and then heated at 100°C for 4min. The samples were cooled at room temperature. A D-xylose standard solution was prepared by dissolving D-xylose in saturated benzoic acid (prepared in deionized water) to obtain 0 mM, 0.7 mM, 1.3 mM, and 2.6 mM, which were consequently added to the color reagent solution alongside the samples. The absorbance of samples and standard solutions at 554nm was determined by using a spectrophotometer (Model 6100, Jenway Ltd., Felsted, Dunmow, CM6 3LB, Essex, England, UK). The standard solution of 0 mmol/L D-xylose was regarded as blank.

### Intestinal redox status

The intestinal samples (~200 mg) were homogenized with cooling saline and then centrifuged at 2,500 rpm for 10 min at 4 °C to collect the supernatant fluid for further assays. The concentration of inducible nitric oxide synthase (iNOS), total nitric oxide synthase (tNOS), hydrogen peroxide (H_2_O_2_), catalase (CAT), glutathione peroxidase (GSH-Px), malondialdehyde (MDA) and superoxide dismutase (SOD) and so on in the intestinal mucosae were determined by using commercially available kits (Nanjing Jiancheng Bioengineering Institute, Nanjing, China).

### qPCR analyses for gene expression in intestine

Each frozen mucosal sample (~100 mg) was powdered under liquid nitrogen using a mortar and pestle, and then homogenized in Trizol buffer. Total RNA was extracted by using the TRIzol Reagent protocol (Invitrogen, Carlsbad, CA, USA). Total RNA was quantified at an OD of 260 nm using the NanoDrop^®^ ND-1000A UV-VIS spectrophotometer (Thermo Scientific, Wilmington, DE, USA), and its purity was evaluated by determining the OD_260_/OD_280_ ratio. When samples showed an OD_260_/OD_280_ = 1.8–2.2, they could be considered 90–100% pure nucleic acids (34). The RNA integrity in each sample was evaluated by using 1% denatured agarose gel electrophoresis. When the sample RNA had a 28S/18S rRNA ratio≥1.8, RNA can be used for quantitative RT-PCR analysis (35). According to the manufacturer's instruction, cDNA was synthesized by using a PrimeScript^®^ RT reagent kit with gDNA Eraser (Takara, Dalian, China) to reverse-transcribe total RNA. And then the synthesized cDNA was stored at −20°C for further analysis.

The primer pairs (Table 1) used to amplify cDNA fragments were used for qPCR as previously described by Yi et al. (34). The SYBR^®^ Premix Ex Taq™ (Takara, Dalian, China) on an Applied Biosystems 7500 Fast Real-Time PCR System (Foster City, CA, USA) was used to perform qPCR. The reaction mixture (50 μL) included 25 μL of SYBR^®^ Premix Ex Taq™ (2 × ), 4 μL of cDNA and 0.2 μM of each primer. Each sample was conducted in triplicate. The reaction conditions of qPCR (two-step amplification): 95°C for 30 s, followed by 40 cycles of 95°C for 5 s and 60°C for 31 s. A subsequent melting curve (9 °C for 15 s, 60°C for 1 min and 95°C for 15 s) with continuous fluorescence measurement and final cooling to room temperature was processed. The melting curves of the products and the size of the amplicons were used to evaluate the specificity of qPCR (36). The ribosomal protein L4 (RPL4) and glyceraldehyde-3-phosphate dehydrogenase (GAPDH) were as references in this study (37). Results were analyzed by 2^−ΔΔ^Ct method (38).

**Table 1:** Sequences of the primers used for quantitative real-time PCR analysis.

**Gene**	**Forward (5^′^-3^′^)**	**Reverse (5^′^-3^′^)**
IGF-1	GCCCAAGGCTCAGAAGG	TTTAACAGGTAACTCGTGC
mTOR	TTGTTGCCCCCTATTG TGAAG	CCTTTCGAGATGGCAATGGA
ALP	CCACTCCCACGTCTTTA CCTTT	CTCTCACCACCCACCACCTT
IL-4	TACCAGCAACTTCGT CCAC	ATCGTCTTTAGCCTTTCCAA
IL-6	TACTGGCAGAAAACA ACCTG	GTACTAATCTGCACAGCCTC
IL-8	TTCGATGCCAGTGCAT AAATA	CTGTACAACCTTCTGCACCCA
IL-10	CGGCGCTGTCATCAATT TCTG	CCCCTCTCTTGGAGCTTGCTA
TNF-α	TCCAATGGCAGA GTGGGTATG	AGCTGGTTGTCTTTCA GCTTCAC
RPL4	GAGAAACCGTCGCCGAAT	GCCCACCAGGAGCAAGTT
GAPDH	CGTCCCTGAGACACG ATGGT	CCCGATGCGGCCAAAT

### Statistical analysis

All data were showed as means ± SD and analyzed using one-way ANOVA. The normality and constant variance for data were tested using Levene's test. Differences among treatment means were determined using Duncan's *post-hoc* tests. All statistical analyses were performed using the SPSS 17.0 software (SPSS, Inc.). *P* < 0.05 was considered to indicate statistical significance.

## Results

### Effects of CCT and CS supplementation on growth performance and DR

Compared with the control group, dietary supplementation with CCT and CS significantly decreased the DRs of weaned piglets (Table 2). Neither CS nor CCT could affect the average daily feed intake (ADFI), average daily gain (ADG), and feed/gain (F/G) of weaned piglets (Table 2).

**Table 2:** Effects of CCT on growth performance and DR of weaned piglets.

**Item**	**Control**	**SC**	**CCT**	***p*-value**
ADFI (kg)	0.66 ± 0.06	0.69 ± 0.10	0.68 ± 0.08	0.767
ADG (kg)	0.39 ± 0.04	0.40 ± 0.06	0.40 ± 0.06	0.878
F/G	1.71 ± 0.07	1.74 ± 0.08	1.73 ± 0.09	0.747
DR (%)	46.6 ± 9.56^a^	6.61 ± 1.58^b^	10.0 ± 2.60^b^	< 0.001

Data are means ± SD, *n* = 6, CS, colistin sulfate; CCT, a complex of cinnamaldehyde, carvacrol and thymol; ADFI, average daily feed intake; ADG, average daily gain; F/G, feed/gain ratio; DR, diarrhea rate. Differences among treatment means were determined using Duncan's *post-hoc* tests. ^a, b^Means within rows with different superscripts differ (*P* < 0.05).

### Effects of CCT and CS supplementation on blood biochemical parameters and hematological parameters in weaned piglets before LPS stimulation

Compared with the control group, dietary supplementation with CCT significantly increased the activity of alanine transaminase (ALT) and the concentrations of cholesterol (CHOL), triacylglycerol (TG), blood urea nitrogen (BUN) and blood chlorine (CL). While dietary supplementation with CCT significantly decreased the concentration of glucose (GLU) and the activity of alkaline phosphatase (ALP) in plasma compared with those of the control group. Dietary supplementation with CS significantly increased the concentration of CL in plasma compared with that of the control group (Table 3). Moreover, compared with the control group, dietary supplementation with CCT significantly elevated the monocytic cell count (MONO), mean platelet volume (MPV) and plateletcrit (PCT) levels in the blood. Dietary CS supplementation significantly elevated (*P* < 0.05) PCT levels in the blood compared with that of the control group (Table 3).

**Table 3:** Effects of CCT supplementation on blood biochemical parameters and hematological parameters in piglets before LPS challenge.

**Items**	**Control**	**CS**	**CCT**	***p*-value**
**Blood biochemical parameters**				
ALT (U/L)	56.8 ± 10.6^a^	65.3 ± 12.5^a^	85.8 ± 7.60^b^	< 0.001
CHOL (mmol/L)	2.21 ± 0.16^a^	2.22 ± 0.29^a^	2.84 ± 0.33^b^	< 0.001
TG (mmol/L)	0.68 ± 0.10^a^	0.56 ± 0.12^a^	0.89 ± 0.16^b^	< 0.001
BUN (mmol/L)	3.12 ± 0.65^a^	2.67 ± 0.35^a^	4.25 ± 0.60^b^	< 0.001
CL (mmol/L)	105 ± 1.27	108 ± 3.35^b^	108 ± 2.16^b^	0.009
GLU (mmol/L)	4.47 ± 0.70^b^	4.79 ± 0.47^b^	3.80 ± 0.50^a^	0.028
ALP (U/L)	327 ± 71.3^b^	328 ± 56.0^b^	240 ± 35.7^a^	0.021
**Hematological parameters**				
MONO (10^9^/L)	0.82 ± 0.13^a^	0.81 ± 0.14^a^	1.36 ± 0.39^b^	< 0.001
MPV (fL)	13.7 ± 2.32^a^	15.6 ± 2.65^ab^	19.9 ± 3.82^b^	0.043
PCT (%)	0.67 ± 0.16^a^	0.99 ± 0.15^b^	1.11 ± 0.31^b^	0.015

Data are means ± SD, n = 6, CS, colistin sulfate; CCT, a complex of Cinnamaldehyde, carvacrol and thymol; ALT, alanine transaminase; CHOL, cholesterol; TG, triacylglycerol; BUN, blood urea nitrogen; CL, blood chlorine; GLU, glucose; ALP, alkaline phosphatase; MONO, monocytic cell count; MPV, mean platelet volume; PCT, plateletcrit. Differences among treatment means were determined using Duncan's *post-hoc* tests. ^a, b^Means within rows with different superscripts differ (*P* < 0.05).

### Effects of CCT and CS supplementation on blood biochemical parameters and hematological parameters in weaned piglets challenged with LPS

Compared with the control group, LPS challenge obviously increased the concentrations of total bilirubin (TBLL), TG, BUN, creatinine (Crea) and CL, and gamma-glutamyl transferase (GGT) activity. LPS challenge significantly decreased the concentration of GLU in the plasma compared with that of the control group. However, dietary supplementation of CCT significantly increased aspartate transaminase (AST) activity and TG level in LPS-challenged weaned pigs. Dietary supplementation of CCT significantly decreased GLU concentration, as well as the activities of GGT and ALP in LPS-challenged weaned pigs (Table 4). Compared with the control group, LPS challenge significantly decreased the levels of white blood cell (WBC), neutrophil count (NEU), lymphocyte count (LYM), MONO, eosinophile count (EOS), basophilic leukocyte count (BASO), and platelet count (PLT). However, dietary supplementation of CCT significantly reduced the number of PLT in the blood in LPS-challenged weaned piglets (Table 4).

**Table 4:** Effects of CCT supplementation on blood biochemical parameters and hematological parameters in weaned piglets after LPS challenge.

**Item**	**Control**	**LPS**	**CS+LPS**	**CCT+LPS**	***p*-value**
**Blood biochemical parameters**					
ALT (U/L)	53.0 ± 11.70^a^	55.7 ± 7.45^ab^	63.3 ± 7.08^ab^	66.8 ± 6.70^b^	0.050
AST (U/L)	40.9 ± 3.41^a^	44.4 ± 9.15^a^	52.3 ± 10.1^a^	68.3 ± 14.2^b^	0.001
TBLL (μmol/L)	3.23 ± 0.71^a^	6.27 ± 1.73^bc^	5.28 ± 1.80^b^	7.86 ± 1.39^c^	< 0.001
TG (mmol/L)	0.49 ± 0.04^a^	0.85 ± 0.27^b^	0.68 ± 0.13^ab^	1.19 ± 0.29^c^	< 0.001
BUN (mmol/L)	1.87 ± 0.10^a^	2.37 ± 0.11^b^	2.35 ± 0.68^b^	2.53 ± 0.14^b^	0.034
Crea (μmol/L)	95.8 ± 6.14^a^	135 ± 14.0^b^	142 ± 33.6^b^	135 ± 20.7^b^	0.005
GLU (mmol/L)	5.36 ± 0.60^c^	3.20 ± 1.19^b^	3.72 ± 1.02^b^	1.98 ± 0.38^a^	< 0.001
CL (mmol/L)	104 ± 1.9^a^	107 ± 0.7^b^	107 ± 2.1^b^	107 ± 1.7^b^	0.007
ALP (U/L)	312 ± 47.2^a^	315 ± 19.2^a^	326 ± 41.1^a^	261 ± 19.3^b^	0.034
GGT (U/L)	36.5 ± 6.0^a^	53.1 ± 9.8^b^	49.5 ± 12.8^b^	35.5 ± 4.7^a^	0.006
**Hematological parameters**					
WBC (10^9^/L)	15.72 ± 2.17^b^	1.35 ± 0.38^a^	1.95 ± 0.88^a^	1.57 ± 0.44^a^	< 0.001
NEU (10^9^/L)	4.99 ± 1.14^b^	0.50 ± 0.22^a^	0.98 ± 0.30^a^	0.73 ± 0.27^a^	< 0.001
LYM (10^9^/L)	3.27 ± 0.85^b^	0.29 ± 0.14^a^	0.27 ± 0.14^a^	0.33 ± 0.10^a^	< 0.001
MONO (10^9^/L)	3.13 ± 0.78^b^	0.43 ± 0.10^a^	0.37 ± 0.32^a^	0.36 ± 0.07^a^	< 0.001
EOS (10^9^/L)	0.22 ± 0.10^b^	0.06 ± 0.02^a^	0.10 ± 0.08^a^	0.05 ± 0.02^a^	0.002
BASO (10^9^/L)	0.62 ± 0.29^b^	0.17 ± 0.09^a^	0.40 ± 0.20^ab^	0.28 ± 0.09^a^	0.005
PLT (10^9^/L)	571 ± 53.8^c^	371 ± 30.2^b^	332 ± 65.0^b^	221 ± 35.3^a^	< 0.001

Data are means ± SD, *n* = 6, CS, colistin sulfate; CCT, a complex of cinnamaldehyde, carvacrol and thymol; ALT, alanine transaminase; AST, aspartate transaminase; TBIL, total bilirubin; TG, triacylglycerol; BUN, blood urea nitrogen; Crea, creatinine; GLU, glucose; CL, blood chlorine; GGT, gamma-glutamyl transferase; WBC, white blood cell; NEU, neutrophil count; LYM, lymphocyte count; MONO, monocytic cell count; EOS, eosinophile count; BASO, basophilic leukocyte count; PLT, platelet count. Differences among treatment means were determined using Duncan's *post-hoc* tests. ^a − c^Means within rows with different superscripts differ (*P* < 0.05).

### Effects of CCT and CS supplementation on intestinal absorption function and stress responses

Compared with the control group, LPS challenge significantly reduced the concentrations of D-Xylose in the plasma. Dietary supplementation with CS tended to reduce the decreased content of D-Xylose in the plasma in LPS-challenged weaned piglets. However, compared with the CS+LPS group, dietary supplementation with CCT obviously increased the decreased content of D-Xylose in the plasma in LPS-challenged weaned piglets (Table 5). Compared with the control group, LPS challenge obviously increased the concentrations of cortisol (COR) and prostaglandin (PG) E2 in plasma. Dietary supplementation with CS significantly reduced the increased concentration of COR in plasma in LPS-challenged weaned piglets. However, dietary supplementation with CCT significantly increased the content of PGE_2_ in the plasma in LPS-challenged weaned piglets (Table 5).

**Table 5:** CCT and CS supplementation on the concentrations of D-xylose, COR (Cortisol), and PGE_2_ (Prostaglandin E2) in the plasma of LPS-challenged piglets.

**Item**	**Control**	**LPS**	**CS+LPS**	**CCT+LPS**	***p*-value**
D-xylose	1.56 ± 0.15^a^	1.14 ± 0.21^bc^	1.31 ± 0.26^b^	0.89 ± 0.17^c^	< 0.001
COR	37.67 ± 6.47^a^	273.72 ± 31.17^c^	216.57 ± 24.37^b^	291.26 ± 54.28^c^	< 0.001
PGE_2_	109.47 ± 7.28^a^	293.86 ± 73.37^b^	280.29 ± 116.44^b^	424.77 ± 123.33^c^	< 0.001

Data are means ± SD, *n* = 6, CS, colistin sulfate; CCT, a complex of cinnamaldehyde, carvacrol and thymol. Differences among treatment means were determined using Duncan's *post-hoc* tests. ^a − c^Means within rows with different superscripts differ (*P* < 0.05).

### Effects of CCT and CS supplementation on the activities of intestinal disaccharidases

**Figure 1:**
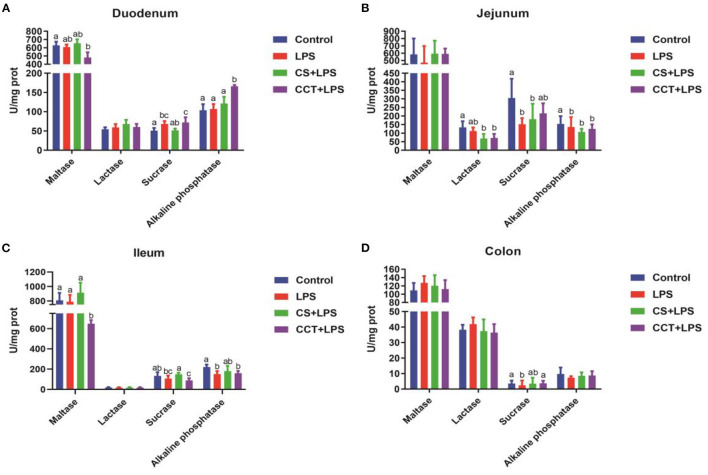
Effects of CCT and CS supplementation on the activities of intestinal disaccharidases in LPS-challenged piglets. The activities of maltase (*p* = 0.062), lactase (*p* = 0.882), sucrase (*p* = 0.004) and alkaline phosphatase (*p* = 0.006) in duodenum **(A)**, the activities of maltase (*p* = 0.440), lactase (*p* = 0.002), sucrase (*p* = 0.003) and alkaline phosphatase (*p* = 0.006) in jejunum **(B)**, the activities of maltase (*p* = 0.002), lactase (*p* = 0.922), sucrase (*p* = 0.005) and alkaline phosphatase (*p* = 0.010) in ileum **(C)**, and the activities of maltase (*p* = 0.741), lactase (*p* = 0.418), sucrase (*p* = 0.099) and alkaline phosphatase (*p* = 0.417) in colon **(D)** in LPS-challenged piglets. Data are means ± SD, *n* = 6, CS, colistin sulfate; CCT, a complex of cinnamaldehyde, carvacrol and thymol. Differences among treatment means were determined using Duncan's *post-hoc* tests. ^a − c^Means within rows with different superscripts different (*P* < 0.05).

Compared with the control group, LPS challenge significantly increased the activity of sucrase in the duodenum (Figure 1A), while significantly reducing the activities of sucrase and alkaline phosphatase in the jejunum (Figure 1B), alkaline phosphatase in the ileum (Figure 1C) and sucrase in the colon (Figure 1D). Dietary supplementation with CS obviously reduced the activity of lactase in the jejunum (Figure 1B) and significantly increased the activity of sucrase in the ileum (Figure 1C). Dietary supplementation with CCT obviously reduced the activity of lactase in the jejunum (Figure 1B) and maltase in the ileum (Figure 1C), and increased the activity of sucrase in the colon (Figure 1D).

### Effects of CCT and CS supplementation on intestinal redox status

**Figure 2:**
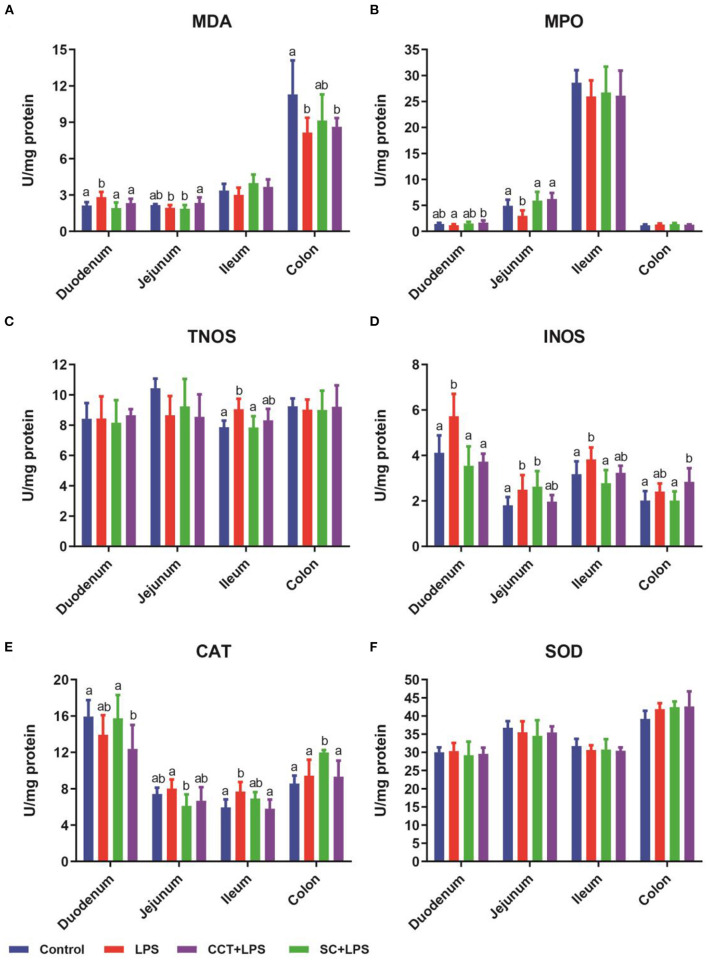
Effects of CCT and CS supplementation on intestinal redox status in LPS-challenged piglets. Data are means ± SD, *n* = 6, CS, colistin sulfate; CCT, a complex of cinnamaldehyde, carvacrol and thymol; malondialdehyde (MDA) in duodenum (*p* = 0.005), jejunum (*p* = 0.042), ileum (*p* = 0.077) and colon (*p* = 0.046) **(A)**; myeloperoxidase (MPO) in duodenum (*p* = 0.055), jejunum (*p* = 0.001), ileum (*p* = 0.649) and colon (*p* = 0.192) **(B)**; total nitric oxide synthase (tNOS) in duodenum (*p* = 0.914), jejunum (*p* = 0.099), ileum (*p* = 0.016) and colon (*p* = 0.965) **(C)**; inducible nitric oxide synthase (iNOS) in duodenum (*p* < 0.001), jejunum (*p* = 0.037), ileum (*p* = 0.016) and colon (*p* = 0.015) **(D)**; catalase (CAT) in duodenum (*p* < 0.048), jejunum (*p* = 0.049), ileum (*p* = 0.006) and colon (*p* = 0.001) **(E)**; superoxide dismutase (SOD) in duodenum (*p* < 0.860), jejunum (*p* = 0.635), ileum (*p* = 0.640) and colon (*p* = 0.120) **(F)**. Differences among treatment means were determined using Duncan's *post-hoc* tests. ^a, b^Means within rows with different superscripts differ (*P* < 0.05).

Compared with the control group, LPS challenge significantly increased the concentration of malondialdehyde (MDA) in the duodenum (Figure 2A) and the activities of total nitric oxide synthase (tNOS) in the ileum (Figure 2C), inducible nitric oxide synthase (iNOS) in the duodenum, jejunum and ileum (Figure 2D) and catalase (CAT) in the ileum (Figure 2E). LPS challenge obviously reduced the concentration of malondialdehyde (MDA) in the colon (Figure 2A) and the activity of myeloperoxidase (MPO) in the jejunum (Figure 2B) compared with those of the control group. Dietary supplementation with CS significantly reduced the concentration of malondialdehyde (MDA) in the duodenum (Figure 2A) and the activities of tNOS in the ileum (Figure 2C) and iNOS in the duodenum and ileum (Figure 2D) and CAT in the ileum (Figure 2E) in LPS-challenged weaned piglets. Dietary supplementation with CS obviously increased the activities of MPO in the jejunum (Figure 2B) and CAT in the colon (Figure 2E) in LPS-challenged weaned piglets. Dietary supplementation with CCT significantly reduced the concentration of MDA in the duodenum (Figure 2A) and the activities of iNOS in the duodenum (Figure 2D) and CAT in the colon (Figure 2E) in LPS-challenged weaned piglets. However, dietary supplementation with CCT significantly increased the concentration of MDA in the jejunum (Figure 2A) and the activities of MPO in the duodenum and jejunum (Figure 2B).

### Effects of CCT and CS supplementation on the mRNA levels of mucosal growth-related, immune and mucosal energy metabolism-related genes in the jejunum

Compared with the control group, LPS challenge significantly decreased the mRNA levels of mucosal growth-related genes [mammalian target of rapamycin (mTOR) and alkaline phosphatase (ALP)], immune-related genes (IL-4, IL-6, IL-8, IL-10 and TNF-α) in the jejunum. Dietary supplementation with CS significantly increased the mRNA levels of mucosal growth-related genes (insulin-like growth factors-1 (IGF-1), mTOR and ALP), immune-related genes (IL-6 and IL-8) in LPS-challenged weaned piglets. Dietary supplementation with CS obviously reduced the mRNA levels of immune-related gene (IL-4) in the jejunum in LPS-challenged weaned piglets. Dietary supplementation with CCT significantly increased the immune-related genes (IL-6 and IL-8) in LPS-challenged weaned piglets. Dietary supplementation with CCT significantly reduced the immune-related gene (IL-4) in the jejunum in LPS-challenged weaned piglets. However, compared with the control group, dietary supplementation with CCT increased immune-related gene (IL-6) more than twice in the jejunum in LPS-challenged weaned piglets (Table 6).

**Table 6:** Effects of CCT supplementation on the mRNA levels of mucosal growth-related genes, immune-related genes and mucosal energy metabolism-related genes in the jejunum in piglets after LPS challenge.

**Item**	**Control**	**LPS**	**CS+LPS**	**CCT+LPS**	***p*-value**
**Mucosa growth related genes**					
IGF-1	1.000 ± 0.213^b^	0.724 ± 0.091^ab^	2.642 ± 0.512^c^	0.488 ± 0.161^a^	< 0.001
mTOR	1.000 ± 0.157^a^	0.583 ± 0.104^b^	0.999 ± 0.178^a^	0.641 ± 0.124^b^	< 0.001
ALP	1.000 ± 0.155^a^	0.674 ± 0.125^b^	1.092 ± 0.159^a^	0.581 ± 0.055^b^	< 0.001
**Immune related genes**					
IL-4	1.000 ± 0.134^a^	0.764 ± 0.095^b^	0.537 ± 0.076^c^	0.357 ± 0.051^d^	< 0.001
IL-6	1.000 ± 0.109^b^	0.561 ± 0.089^a^	1.295 ± 0.256^c^	2.022 ± 0.264^d^	< 0.001
IL-8	1.000 ± 0.154^a^	0.473 ± 0.069^d^	0.655 ± 0.117^c^	0.831 ± 0.164^b^	< 0.001
IL-10	1.000 ± 0.168^a^	0.717 ± 0.077^b^	0.885 ± 0.169^ab^	0.861 ± 0.149^ab^	0.026
TNF-α	1.000 ± 0.140^a^	0.499 ± 0.067^b^	0.584 ± 0.102^b^	0.497 ± 0.051^b^	< 0.001

Data are means ± SD, *n* = 6, CS, colistin sulfate; CCT, a complex of cinnamaldehyde, carvacrol and thymol; IGF-1, insulin-like growth factors-1; mTOR, mammalian target of rapamycin; ALP, alkaline phosphatase. Differences among treatment means were determined using Duncan's *post-hoc* tests. ^a − d^Means within rows with different superscripts differ (*P* < 0.05).

### Effects of CCT and CS supplementation on the mRNA levels of immune related genes in mesenteric lymph nodes

Compared with the control group, LPS challenge obviously decreased the mRNA of immune-related genes (IL-4, IL-6, IL-8, IL-10, and TNF-α) in mesenteric lymph nodes in LPS-challenged weaned piglets. Dietary supplementation with CS significantly increased the mRNA levels of immune-related genes (IL-4, IL-6, IL-8, and IL-10) in mesenteric lymph nodes in LPS-challenged weaned piglets. Dietary supplementation with CCT also significantly increased the mRNA levels of immune-related genes (IL-4, IL-6, IL-8, and IL-10) in mesenteric lymph nodes in LPS-challenged weaned piglets. However, compared with the control group, dietary supplementation with CCT increased the immune-related gene (IL-6) by more than 1.7 in the jejunum in LPS-challenged weaned piglets (Table 7).

**Table 7:** Effects of CCT supplementation on the mRNA levels of immune related gene in mesenteric lymph nodes in piglets after LPS challenge.

**Items**	**control**	**LPS**	**CS+LPS**	**CCT+LPS**	***P*-value**
IL-4	1.000 ± 0.242^a^	0.184 ± 0.048^c^	0.447 ± 0.205^b^	0.425 ± 0.196^b^	< 0.001
IL-6	1.000 ± 0.139^b^	0.618 ± 0.084^a^	1.193 ± 0.296^b^	1.772 ± 0.388^c^	< 0.001
IL-8	1.000 ± 0.230^c^	0.455 ± 0.157^a^	0.704 ± 0.089^b^	0.996 ± 0.287^c^	< 0.001
IL-10	1.000 ± 0.092^b^	0.692 ± 0.065^a^	1.098 ± 0.200^b^	1.618 ± 0.403^c^	< 0.001
TNF-a	1.000 ± 0.116^a^	0.606 ± 0.121^b^	0.596 ± 0.188^b^	0.704 ± 0.105^b^	< 0.001

Data are means ± SD, *n* = 6, CS, colistin sulfate; CCT, a complex of cinnamaldehyde, carvacrol and thymol. Differences among treatment means were determined using Duncan's *post-hoc* tests. ^a − c^Means within rows with different superscripts differ (*P* < 0.05).

## Discussion

To solve various problems encountered in weaned piglet feeding, a variety of green, safe and efficient antibiotic substitutes that can reduce the DR and increase the growth performance in weaned piglets have been studied. Dietary supplementation with 50 mg/kg oleum cinnamomi could increase ADFI and reduce DR in piglets (34). Yang et al. (16) found that 400 mg/d or 800 mg/d cinnamic aldehyde could increase feed intake, but have no effect on the average daily gain in feedlot cattle. Dietary supplementation with *Lactobacillus casei* decreased the F/G and DR in weaned piglets (35). N-Acetylcysteine supplemented in diet did not reduce DR and affect the growth performance in weaned piglets, but N-acetylcysteine improved intestinal function in LPS-challenged piglets (33). Glutamate precursor α-ketoglutarate supplemented in deity improved LPS-induced liver injury by improving energy metabolism and increasing anti-oxidative capacity in LPS-challenged young pigs (13). These studies suggested that potential antibiotic substitutes supplementation may play different roles in improving the growth performance of weaned piglets. In this study, our results showed that dietary supplementation with CCT reduced the DR of weaned piglets. Meanwhile, dietary supplementation with CS (as positive control) also reduced the DR of weaned piglets (Table 2). Previous study reported that CS probably will be the 'last-line' therapeutic drug against multidrug-resistant Gram-negative pathogens in the 21st century (39). CS, as an antibiotic growth promoter, had been broadly used as a growth-promoting agent in feed to improve growth performance and attenuate gastrointestinal infections in weaned piglets (28, 29, 40). However, CS as a feed additive has been banned to use in animal diets in China since May 2017 (41). Therefore, it is feasible and meaningful to use CS as a positive control to evaluate the effectiveness of novel alternatives of antibiotic feed additives. Our results showed that CCT supplementation exerted the same effect as CS in reducing the DR in weaned piglets, suggesting that CCT may be a promising alternative to antibiotic feed additives in piglets.

Although dietary supplementation with CCT reduced the DR in weaned piglets, it is still unclear the effects of CCT supplementation on physiological function and the ability to respond to external stimuli in weaned piglets. It is an important role for blood biochemical and blood cell indicators to reflect the status of health, cell permeability, and the level of metabolism (2). The changes in blood biochemical and blood cell indicators can reflect the function of multiple tissues and organs in the body. Plasma ALT, AST, TBIL, ALP and GGT activities are considered to be non-specific and specific markers for hepatic injury (42). Plasma Crea, CL and BUN levels can be associated with renal function (43). CHOL is involved in the stability of many cell membranes in the body, including the synthesis of sex hormones and vitamin D (44). Serum glucose concentrations can reflect hormone secretion levels in the body (2). Blood MONO can respond to the body's immune levels (2). Blood MPV and PCT are indicators that respond to hematologic diseases. In this study, compared with the control group, CCT supplementation significantly changed multiple blood biochemical parameters (ALT, CHOL, TG, BUN, CL, GLU and ALP) and hematological parameters (MONO, MPV and PCT), however, CS supplementation only changed blood biochemical parameters (CL) and hematological parameters (PCT) (Table 3). These results suggested that CCT supplementation may not be as good as CS in improving physiological function in weaned piglets.

The effects of CCT supplementation on blood biochemical and hematological parameters in LPS-challenged piglets were further evaluated. Consistent with previously reported results (2, 33, 35, 45) that LPS challenge changed the blood biochemical and hematological parameters in piglet, our results showed that blood biochemical parameters (TBLL, TG, BUN, CL, Crea, GLU, and GGT) and hematological parameters (WBC, NEU, LYM, MONO, EOS, BASO, and PLT) were significantly changed in LPS-challenged weaned piglets. Previous studies showed that the porcine liver function may be impaired based on the elevations in the activities of plasma AST and GGT under LPS stimulation (33, 35). The increases in AST and GGT activity in plasma may indicate liver damage by LPS challenge. TG is an important source of energy for the body, and when the TG in the body is too high, it will continue to accumulate fat in the liver, resulting in fatty liver (46). Blood GLU is the most direct source of energy for various life activities, the level of blood GLU concentration directly reflects the vitality of animals in the normal blood GLU range (47). LPS stimulation can cause the liver to be suppressed in the gluconeogenesis pathway, resulting in persistent hypoglycemia *in vivo*. The main function of PLT is coagulation and hemostasis, and it play an important role in the process of hemorrhagic coagulation (48). Our results showed that CS supplementation did not significantly alter the blood biochemical indicators and hematological parameters in piglets challenged with LPS stimulation (Table 4). While CCT supplementation significantly reduced the increase in GGT activity in LPS-challenged weaned piglets, CCT supplementation exacerbated changes in multiple indicators in LPS-challenged weaned piglets, including the decrease in GLU and PLT and the increase in ALT activity and TBLL levels. These results suggested that CCT supplementation may increase the susceptibility of weaned piglets to LPS challenge.

The above findings suggested that CCT supplementation may have a negative effect on body homeostasis in weaned piglets. However, how CCT supplementation reduced the DR in weaned piglets remains to be further studied. Consistent with previous reports that LPS challenge can induce intestinal oxidative stress in weaned piglets (2, 33, 35, 45), these results in this study showed that LPS challenge significantly increased the activities of tNOS and iNOS in the ileum and iNOS in the duodenum and jejunum and the concentration of MDA in the duodenum. MDA is usually used to assess the degree of damage to the cell membranes (2). MPO is an important iron-containing lysosome in neutrophils and monocytes, and plays an important role in the body's immune defense process (2). Inducible nitric oxide synthase (iNOS) delivers a great amount of NO compared to the constitutive isoforms of NOS (49). Physiological concentrations of NO play a crucial role in the maintenance of intestinal mucosal integrity, but pathological levels are detrimental to the gut (50, 51). CS supplementation ameliorated the adverse effects of LPS challenge on intestinal oxidative responses in weaned piglets (Figure 2). Furthermore, CCT supplementation also ameliorated the adverse effects of LPS challenge on intestinal oxidative responses in weaned piglets. These results suggested that CCT supplementation improved the antioxidant capacity in LPS-challenged weaned piglets. CCT supplementation may reduce the DR in piglets by increasing the antioxidant capacity.

Fifty to eighty percent of the ingredients in the animal diet are carbohydrates, and the energy required for animal growth mainly comes from carbohydrates (52–54). The carbohydrates in the diet are composed of polysaccharides, oligosaccharides, and monosaccharides (52). Only monosaccharides can be directly absorbed by the intestine (55). However, polysaccharides and oligosaccharides need to be decomposed into disaccharides by a series of enzymes, and then the disaccharides are then decomposed into monosaccharides by disaccharidases in the mucous membrane before they can be absorbed and utilized (33, 52–54, 56, 57). Therefore, disaccharidases play a key role in the absorption and utilization of carbohydrates. In this study, LPS challenge decreased the activities of sucrase in the jejunum and colon and the activities of alkaline phosphatase in the jejunum and ileum (Figure 1). These results suggested that LPS challenge reduced the intestinal function of piglets by reducing the activity of disaccharidases. CS supplementation increased the activities of sucrase in the jejunum and colon, suggesting that CS may improve intestinal function in LPS-challenged piglets. However, although CCT supplementation ameliorated the adverse effects of LPS challenge on sucrase activity in the colon, CCT supplementation exacerbated the LPS-induced decrease in the activity of maltase in the ileum. These results suggested that the effect of CCT supplementation on intestinal absorptive function needs to be further investigated.

The plasma D-xylose absorbed from the intestinal lumen is a useful indicator for intestinal absorption capacity and mucosal integrity (58, 59). D-xylose is readily absorbed by the jejunum in healthy piglets. However, under LPS stimulation or malabsorption, D-xylose is not absorbed by the intestine, thus reducing the D-xylose content in plasma (30). In this study, LPS challenge decreased the concentration of D-xylose in plasma, suggesting that LPS challenge reduced the intestinal absorption function (Table 5). Zhou et al. (60) found that cinnamaldehyde can improve absorption capacity in grass carp. Consistent with previously reported results, CS supplementation had a tendency to increase the content of D-xylose in plasma in LPS-challenged weaned piglets. However, CCT supplementation had a tendency to reduce the concentration of D-xylose in plasma in LPS-challenged weaned piglets. Meanwhile, CCT supplementation had no effect on the morphology and structure of the jejunum in LPS-challenged weaned piglets (data not shown). These results suggested that CCT supplementation exacerbated the decrease of intestinal absorption function in LPS-challenged weaned piglets, however, the mechanism remains unclear.

Combined with the above results, we speculated that CCT supplementation aggravated the decline in intestinal absorption function in LPS-challenged weaned piglets by altering the intestinal stress responses. Stress response, including activation of the sympathetic nervous system, glucocorticoid secretion, and changes in emotional behaviors, is a state of physiological or psychological stress caused by adverse stimuli (61). COR is a glucocorticoid that is widely used as a biomarker for the detection of stress responses in pigs (62). PGE_2_ is involved in these stress responses mentioned above and it is released from various cell types depending on the type of stressors present and acts in the vicinity of its synthesis (61). Previous studies reported that LPS challenge significantly increased the concentrations of COR and PGE_2_ in plasma (54, 63). Consistent with previous reports, our results showed that LPS challenge increased the concentrations of COR and PGE_2_ in plasma in pigs, suggesting that LPS challenge elicited a stress response in piglets (Table 5). CS supplementation reduced the concentration of COR in plasma of LPS-challenged weaned piglets. However, CCT supplementation increased the concentration of PGE_2_ in plasma of LPS-challenged weaned piglets. These results indicated that CS supplementation alleviated the stress response in LPS-challenged weaned piglet, whereas CCT supplementation exacerbated the stress response in LPS-challenged weaned piglet by increasing the concentration of PGE_2_.

PGE_2_ is involved in various aspects of inflammation and immunity stress (61). PGE_2_, as a mediator, functions in immune inflammation (64). PGE_2_ as a key molecule regulates the activation, maturation, migration, and cytokine secretion of innate immune cells including natural killer cells, dendritic cells, neutrophils, and macrophages (65). In this study, CCT supplementation significantly increased the levels of pro-inflammatory factor IL-6 in the jejunum and mesenteric lymph nodes in LPS-challenged weaned piglets, suggesting that CCT supplementation may exacerbate the intestinal dysfunction by altering the immune stress response in LPS-challenged weaned piglets.

In addition, ALP in the intestine tract is associated with the proliferation and differentiation of intestinal epithelial cell, so ALP is regarded as a critical biomarker enzyme for changes in the primary digestive and absorptive functions of the small intestine (66). The mTOR signaling pathway plays a vital role in enterocyte growth, proliferation, and regeneration (67, 68), and thereby being conducive to the recovery of the small intestinal mucosa after damage (69, 70). The IGF-I plays a prominent role in regulating cell metabolism, proliferation, and differentiation (71, 72). Consistent with the previous study by Yi et al. (23), gene expressions of ALP, mTOR, and IGF-I in the jejunum were dramatically decreased by LPS challenge. In the study, CS significantly increased the mRNA levels of ALP, mTOR, and IGF-I in the jejunum in LPS-challenged weaned piglets, whereas CCT didn't affect mRNA levels for ALP, mTOR, and IGF-I in LPS-challenged piglets. These results indicated that CS improved jejunal mucosal growth in LPS-challenged weaned piglets, whereas CCT did not.

## Conclusions

In conclusion, dietary supplementation with CCT reduced the DR in weaned piglets and alleviated intestinal oxidative stress in LPS-challenged weaned piglets. However, CCT supplementation tended to aggravate the intestinal absorption dysfunction in LPS-challenged weaned piglets by altering the immune stress response. Furthermore, CCT was less effective than CS in improving intestinal function in LPS-challenged weaned piglets. Therefore, our results suggest whether CCT can be as an effective feed additive still needs further study for weaned piglets.

## Data availability statement

The original contributions presented in the study are included in the article/supplementary material, further inquiries can be directed to the corresponding author.

## Ethics statement

The animal study was reviewed and approved by the Animal Care and Use Committee at Wuhan Polytechnic University (2014-0514, May 10, 2014).

## Author contributions

YH designed the study and revised the manuscript. YZ, QL, and YD wrote and revised the manuscript. ZW, LW, DZ, and QL collected and analyzed experimental results. QL, DY, TW, and ZW participated in the discussion of results and the revision of the paper. All authors contributed to the data interpretation and approved the final version of the manuscript.
